# Obliteration of Intercondylar Notch Mimicking Flexion-Extension Gap Imbalance in a Cruciate Retaining Total Knee Arthroplasty

**DOI:** 10.1155/2015/716148

**Published:** 2015-06-22

**Authors:** Harun Resit Gungor, Esat Kiter, Semih Akkaya, Nusret Ok, Cagdas Yorukoglu

**Affiliations:** Orthopedics and Traumatology Department, Pamukkale University Medical Faculty, Pamukkale, 20070 Denizli, Turkey

## Abstract

Following total knee arthroplasty (TKA), the most frequent cause of extension deficit and limitation of range of motion in early postoperative period is related to improper tensioning of soft tissues and failure to balance extension and flexion gaps. If a cruciate retaining (CR) prosthesis is the planned implant, then attention should be given to balancing the posterior cruciate ligament (PCL), and any factor that alters this balance may also cause deterioration of knee balance in postoperative period. Here, we report on an unusual case referred from another hospital because of continuous pain and restriction of knee motion in early postoperative period following CR-designed TKA that was initially thought to be due to flexion-extension imbalance. However, during the revision procedure, extruded cement to the intercondylar notch was found to be both mechanically blocking terminal extension and limiting flexion by possible mechanism of irritation of the synovial nerve endings around the stretched anterior fibers of PCL during flexion. This case was successfully treated by removal of extruded cement from intercondylar notch to decompress PCL, polyethylene exchange, and secondary patellar resurfacing.

## 1. Introduction

Many potential causes may lead to restriction of motion and painful total knee arthroplasty (TKA) in the absence of infection during the early postoperative period. The most frequent causes of extension deficit and limitation of range of motion in early postoperative period are related to improper tensioning of soft tissues, rotational malalignment of the components, and failure to balance extension and flexion gaps [1–3].

During the operation, following bone resection and soft tissue balancing to correct the preoperative axial deformity and contractures, all the osteophytes including posterior ones are removed [4]. If a cruciate retaining (CR) prosthesis is the planned implant, then attention should be given to balancing the PCL, and rectangular gap both in flexion and in extension along with proper rotational alignment of components should be obtained at the end [5, 6]. In case of any residual contracture, then it may be necessary to further release the posterior capsule or resect additional distal femur [4, 6]. During the trial of the implants, the knee must come to full extension, and residual flexion contracture should not be accepted since this will not correct over the time [6]. However, in the postoperative period obliteration of intercondylar notch may mimic clinical signs of flexion-extension mismatch either by mechanically blocking extension or by stimulating nerve endings in synovia around the PCL in CR-designed TKA or both [7, 8]. Therefore, the aim of this report is to show that obliteration of intercondylar notch may mimic signs of soft tissue disequilibrium in CR-designed TKA patients although this is a rare entity.

Here, we report on an unusual case referred from another hospital because of continuous pain and restriction of knee motion in early postoperative period following CR-designed TKA that was initially thought to be due to flexion-extension gap imbalance. The patient and her family were informed that data from the case would be submitted for publication and gave their consent.

## 2. Case Report

**Figure 1:**
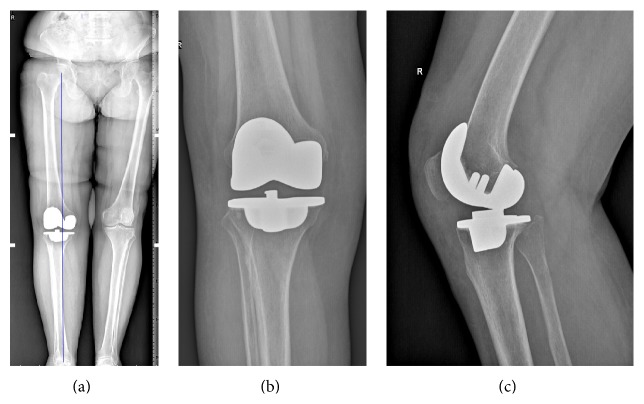
Long standing anteroposterior X-ray showing adequate anatomic alignment (a) and anteroposterior and lateral nonweight bearing radiographs ((b) and (c), resp.).

A 55-year-old woman with a known history of osteoarthritis who has undergone right total knee arthroplasty in another clinic four months ago was referred to our hospital. She was unable to walk due to limitation of her knee motion with persistent pain unresolved despite postoperative intense physical therapy and exercise program. The physical examination of the patient demonstrated insignificant swelling but no effusion in her knee, and the active range of motion was 70° of flexion and 10° of extension deficit. Her neurovascular examination was normal. Western Ontario and McMaster Universities Osteoarthritis Index (WOMAC) score was 38 and visual analogue scale score (VAS) was 8 (0 best, 10 worst). There were no signs of infection clinically, and serum laboratory analyses were within reference values. From her hospital track records, it was learned that mobile insert CR-designed TKA (NexGen, Warsaw, IN) was implanted without surgical difficulty. Her radiographs showed that the femoral and tibial components were properly implanted with slight varus thrust, and the alignment patella was adequate (Figure 1). Reflex sympathetic dystrophy was thought to be unlikely and computerized tomography (CT) of the components to examine rotational alignment of the components showed insignificant findings.

**Figure 2:**
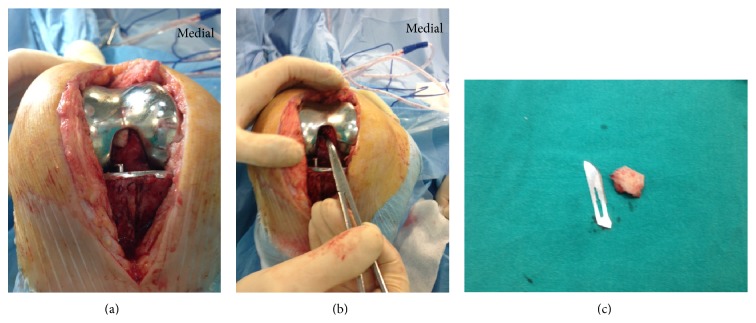
A large cement part extruded into intercondylar notch impinging PCL was seen intraoperatively (a), following removal of cement, PCL was relaxed (b), and the removed part of cement is seen on (c).

Examination of range of motion under anesthesia revealed improvement of flexion to 90° but 10° of extension deficit remained the same. Revision of the components was planned to correct flexion and extension gap imbalance and for secondary patellar resurfacing. However, intraoperatively, a large cement part extruded into intercondylar notch impinging PCL and limiting terminal extension was detected and removed (Figure 2). Following removal of the cement, polyethylene insert exchanged with the same size and thickness, and secondary patellar resurfacing was also performed though the patellar cartilage was uniform and congruent.

Her postoperative course was steady after the surgery. The patient allowed weight bearing, and passive and active ROM exercises were begun immediately in early postoperative period. Flexion angle of 90 degrees was achieved at 3rd postoperative day and she was discharged from hospital with a VAS score of 4. At second year follow-up examination WOMAC score was 87, and she was pain-free walking without crutches and climbing stairs. The ROM was 0°–100° of flexion.

## 3. Discussion

Restriction of knee motion and painful total knee arthroplasty in the absence of infection during the early postoperative period are a challenging problem. Rotational malalignment of the components and soft tissue imbalance along with flexion-extension gap mismatch are the most probable causes of pain and limitation of range of motion [1–3].

Most of the time, extension gap is initially addressed by resection of the distal femoral and proximal tibial articular surfaces. A spacer block is then inserted into the extension gap to evaluate alignment of the extremity and soft tissue balance. If one of these factors is not optimal, additional soft tissue release or in some cases additional bony resections are performed [6]. Following this procedure, flexion gap is equalized and proper rotational alignment of femoral component is determined. If residual flexion-extension disequilibrium is not corrected during the index procedure, this problem presents itself in the form of either flexion instability or extension deficit [6]. In our case, extruded cement part was found to be mechanically occluding terminal extension and indentation of PCL was detected at the corresponding part of PCL close to insertion point to anteromedial wall of lateral femoral condyle. The possible mechanism that we assumed to be responsible for the limitation of flexion was the stimulation of the nerve endings of synovia around the stretched anterior part of PCL [7, 9–11]. Findings of the report by Girgis et al. [12] support this mechanism that anterior portion of PCL is stretched in flexed position.

There have been a few cases reported in the literature following TKA obliterating intercondylar notch. Nakamura et al. [7] reported two cases in which hypertrophic synovial tissues obliterating intercondylar notch caused impingement of PCL in CR-designed TKA and resulted in limitation of extension. Their cases were patients with rheumatoid arthritis and the symptoms were developed in subacute fashion. In addition, Carro and Suarez [8] have also reported a similar complication in a primary osteoarthritic knee in which a PS-designed TKA was applied. In their cases, a thickened fibrotic band was trapped in intercondylar notch at 25 degrees flexion between prosthetic trochlea and the tibial polyethylene post. In the literature, it was also reported that ganglions, synovial cysts, and lipomas localized in intercondylar notch might restrict knee motion and cause pain by impinging cruciate ligaments even in intact knees [9–11].

**Figure 3:**
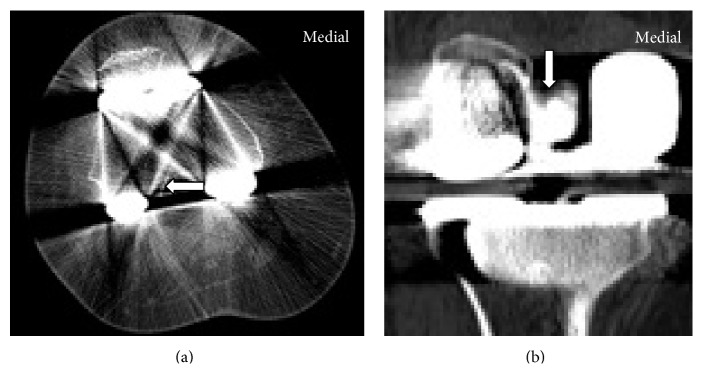
Cement in intercondylar notch (arrow) on CT images.

During application of components excess cement is removed around the prostheses in 3 to 5 minutes of rush period. Following this application, mostly, the components are pressurized by extending the knee while paying attention to avoid forceful hyperextension and potential areas of extrusion for cement are out of sight. Therefore, as a rule, all experienced orthopedic surgeons recheck excess cement around the prosthesis following consolidation of cement. At this point, failure to recheck the extruded cement around the component, though it is an extremely rare condition, may result in compression of neighborhood structures around the knee and may also cause pain and limitation of motion. In these cases, extruded cement parts, most of the time, are clearly visible on plain radiographs. Otani et al. [13] reported on a patient with extruded cement at posterolateral part of tibial tray following TKA. Extruded cement was detected on plain radiographs impinging fibular head, and this case was successfully treated with arthrotomy and removal of the cement part. Karataglis et al. [14] reported on a patient with a cement extrusion into the posteromedial aspect of the knee after a unicondylar knee arthroplasty causing impingement and pain in full extension. They removed cement extrusion arthroscopically from the posterior aspect of the knee. However, in our case intercondylar localization of extruded cement obscured visualization in postoperative plain radiographs and CT images. Therefore, we did not plan arthroscopic intervention preoperatively. The CT images of the patient's knee were retrospectively reevaluated, but the extruded cement was actually not clearly visible due to metallic artifacts (Figure 3).

Finally, we want to emphasize that limitation of extension and painful flexion may be related to obliteration of intercondylar space mimicking flexion-extension disequilibrium in the postoperative period especially in the CR-designed TKA. Although it is an extremely rare condition, extruded cements, synovial proliferations, ganglion cysts, lipomas, and fibrous bands may cause this situation [7–11].
